# (1-Adamant­yl)(4-amino­phen­yl)methanol

**DOI:** 10.1107/S1600536809012987

**Published:** 2009-04-10

**Authors:** Michal Rouchal, Marek Nečas, Robert Vícha

**Affiliations:** a Department of Chemistry, Faculty of Technology, Tomas Bata University in Zlin, Nám. T. G. Masaryka 275, Zlín,762 72, Czech Republic; b Department of Chemistry, Faculty of Science, Masaryk University in Brno, Kamenice 5, Brno–Bohunice, 625 00, Czech Republic

## Abstract

In the racemic crystal of the title compound, C_17_H_23_NO, enanti­omers of the two crystallographically independent mol­ecules are linked into face-to-face *RS*dimers *via* inter­molecular O—H⋯N hydrogen bonds and π–π inter­actions with centroid–centroid distances of 3.7610 (2) Å. The mol­ecules adopt slightly different conformations and contain an adamantane cage consisting of three fused cyclo­hexane rings in almost ideal chair conformations, with C—C—C angles varying within the range 107.2 (4)–111.4 (4)°. In the hydrogen-bonded pair, the benzene rings are almost coplanar, the dihedral angle between them being 1.29 (13)°. The mol­ecular packing in the crystal is stabilized by additional inter­molecular N—H⋯O hydrogen bonds.

## Related literature

The title compound was prepared according to a modification of the procedure of Adkins & Billica (1948 ▶). For some important properties of adamantane-bearing compounds, see: Cromwell *et al.* (1985 ▶), van Bommel *et al.* (2001 ▶).
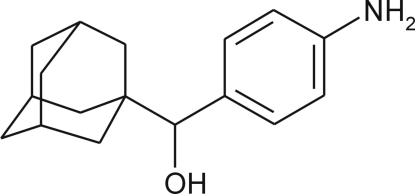


## Experimental

### 

#### Crystal data

C_17_H_23_NO
*M* *_r_* = 257.36
Monoclinic, 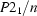

*a* = 8.8107 (5) Å
*b* = 12.1593 (6) Å
*c* = 26.6047 (16) Å
β = 93.046 (5)°
*V* = 2846.2 (3) Å^3^
*Z* = 8
Mo *K*α radiation
μ = 0.07 mm^−1^
*T* = 120 K
0.40 × 0.40 × 0.10 mm

#### Data collection

Kuma KM-4 CCD diffractometer
Absorption correction: none
20748 measured reflections
5001 independent reflections
3444 reflections with *I* > 2σ(*I*)
*R*_int_ = 0.039

#### Refinement

*R*[*F* ^2^ > 2σ(*F* ^2^)] = 0.094
*wR*(*F* ^2^) = 0.270
*S* = 1.19
5001 reflections
359 parameters
H atoms treated by a mixture of independent and constrained refinement
Δρ_max_ = 0.49 e Å^−3^
Δρ_min_ = −0.45 e Å^−3^

### 

Data collection: *CrysAlis CCD* (Oxford Diffraction, 2006 ▶); cell refinement: *CrysAlis CCD*; data reduction: *CrysAlis RED* (Oxford Diffraction, 2006 ▶); program(s) used to solve structure: *SHELXS97* (Sheldrick, 2008 ▶); program(s) used to refine structure: *SHELXL97* (Sheldrick, 2008 ▶); molecular graphics: *ORTEP-3* (Farrugia, 1997 ▶) and *Mercury* (Macrae *et al.*, 2008 ▶); software used to prepare material for publication: *SHELXL97*.

## Supplementary Material

Crystal structure: contains datablocks global, I. DOI: 10.1107/S1600536809012987/lh2796sup1.cif

Structure factors: contains datablocks I. DOI: 10.1107/S1600536809012987/lh2796Isup2.hkl

Additional supplementary materials:  crystallographic information; 3D view; checkCIF report

## Figures and Tables

**Table 1:** Hydrogen-bond geometry (Å, °)

*D*—H⋯*A*	*D*—H	H⋯*A*	*D*⋯*A*	*D*—H⋯*A*
O2—H2*A*⋯N1	0.84	2.06	2.890 (5)	168
O1—H1*A*⋯N2	0.84	2.04	2.876 (5)	171
N1—H1*B*⋯O1^i^	0.83 (6)	2.15 (6)	2.941 (5)	162 (5)
N2—H2*B*⋯O2^ii^	0.91 (7)	2.05 (7)	2.932 (5)	163 (6)

Symmetry codes: (i) 
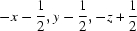
; (ii) 
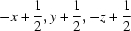
.

**Fig. 1.**
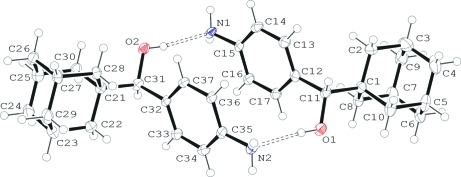
ORTEP of the asymmetric unit with atoms represented as 50% probability ellipsoids and H atoms are shown as small spheres at arbitrary radii.

**Fig. 2.**
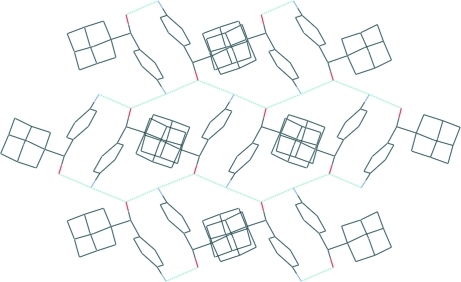
The hydrogen bond cross-linkage in the three-dimensional framework, viewed along the c axis, is drawn by dotted lines. Hydrogen atoms have been omitted for enhanced clarity.

## supplementary crystallographic information

### Comment

The title molecule belongs in the family of promising compounds destined for drugs improvement. Two contrary properties playing significant role in drug design may be modulated by introduction of suitable adamantane-bearing building block into the molecule. The lipophilic adamantane cage itself may increase solubility in non-polar systems (*e.g.* cell membranes), whereas the solubility in polar medium may be enhanced by the formation of non-covalent inclusion complex of adamantane cage with cyclodextrins (Cromwell *et al.* (1985), van Bommel *et al.* (2001)).

The selected asymmetric unit consists of enantiomers of two crystallographically independent molecules with slightly variant in geometries (Fig. 1). Both benzene rings are essentially planar with the maximum deviations from the best planes being 0.008 (4) Å for atom C16 in the first enantiomer and 0.012 (5) Å for atom C33 in the second one. The orientation of the benzene rings is almost coplanar with the dihedral angle between them being 1.29 (13)°. The torsion angles C21–C31–C32–C37 and C1–C11–C12–C17 are -89.7 (5) and 89.5 (5)° respectively. The two enantiomers are linked into pairs *via* two O1–H1A···N2 and O2–H2A···N1 hydrogen bonds (Table 1). Face-to-face π-π interactions stabilize pairs of enantiomers with the centroid-to-centroid distances of 3.7610 (2) Å (*Cg*1 and *Cg*2 are the centroids of the C12–C17 and C32–C37 respectively). Further N2–H2B···O2 and N1–H1B···O1 hydrogen bonds (Table 1, Fig. 2) cross-link the molecules forming the three-dimensional framework.

### Experimental

The title compound was prepared according to a modified literature procedure (Adkins & Billica, 1948). 1-Adamantyl-(4-nitrophenyl)methanol (0.35 mmol, 100 mg) was dissolved in 2 cm^3^ of dioxane and large excess of Raney nickel was added in one portion to this solution. The reaction mixture was vigorously stirred under H_2_ atmosphere at room temperature. After the consumption of all starting material (according to TLC), the mixture was diluted with 5 cm^3^ of water. The water layer was sequentially washed five times with 10 cm^3^ of diethyl ether. The combined organic layers were dried over sodium sulfate and evaporated in vacuum. After the purification of crude product by column chromatography (silica gel; petroleum ether/ethyl acetate, *v*/*v*, 1/1), the desired product was obtained as a pale yellow crystalline powder (88.3 mg, 98%, mp 143–146°C). The single crystals suitable for X-ray analysis were grown by spontaneous evaporation from deuterochloroform at room temperature.

### Refinement

Although several methods, solvents and conditions for crystal growth were tested, the best obtained sample consisted of poor quality crystals affording only a low quality data set. Thus the precision of refined parameters is lowered accordingly. The analysis of the most disagreeable reflections suggested no significant systematic trends which could be attributed to twinning (application of a twin law provided merely negligible improvement in precision) or experimental failures. The H atoms were constrained using standard *SHELXL* facilities with the exceptions of NH_2_ H atoms which were positioned from the diference Fourier map and refined fully.

### Crystal data

**Table:**

C_17_H_23_NO	*F*(000) = 1120
*M**_r_* = 257.36	*D*_x_ = 1.201 Mg m^−^^3^
Monoclinic, *P*2_1_/*n*	Melting point: 145 K
Hall symbol: -P 2yn	Mo *K*α radiation, λ = 0.71073 Å
*a* = 8.8107 (5) Å	Cell parameters from 5001 reflections
*b* = 12.1593 (6) Å	θ = 2.9–25.0°
*c* = 26.6047 (16) Å	µ = 0.07 mm^−^^1^
β = 93.046 (5)°	*T* = 120 K
*V* = 2846.2 (3) Å^3^	Block, white
*Z* = 8	0.40 × 0.40 × 0.10 mm

### Data collection

**Table:**

Kuma KM-4 CCD diffractometer	3444 reflections with *I* > 2σ(*I*)
Radiation source: fine-focus sealed tube	*R*_int_ = 0.039
graphite	θ_max_ = 25.0°, θ_min_ = 2.9°
Detector resolution: 0.06 pixels mm^-1^	*h* = −10→9
ω scans	*k* = −13→14
20748 measured reflections	*l* = −30→31
5001 independent reflections	

### Refinement

**Table:**

Refinement on *F*^2^	Primary atom site location: structure-invariant direct methods
Least-squares matrix: full	Secondary atom site location: difference Fourier map
*R*[*F*^2^ > 2σ(*F*^2^)] = 0.094	Hydrogen site location: inferred from neighbouring sites
*wR*(*F*^2^) = 0.270	H atoms treated by a mixture of independent and constrained refinement
*S* = 1.19	*w* = 1/[σ^2^(*F*_o_^2^) + (0.0778*P*)^2^ + 11.8176*P*] where *P* = (*F*_o_^2^ + 2*F*_c_^2^)/3
5001 reflections	(Δ/σ)_max_ < 0.001
359 parameters	Δρ_max_ = 0.49 e Å^−^^3^
0 restraints	Δρ_min_ = −0.45 e Å^−^^3^

### Special details

**Table:**

Geometry. All e.s.d.'s (except the e.s.d. in the dihedral angle between two l.s. planes) are estimated using the full covariance matrix. The cell e.s.d.'s are taken into account individually in the estimation of e.s.d.'s in distances, angles and torsion angles; correlations between e.s.d.'s in cell parameters are only used when they are defined by crystal symmetry. An approximate (isotropic) treatment of cell e.s.d.'s is used for estimating e.s.d.'s involving l.s. planes.
Refinement. Refinement of *F*^2^ against ALL reflections. The weighted *R*-factor *wR* and goodness of fit *S* are based on *F*^2^, conventional *R*-factors *R* are based on *F*, with *F* set to zero for negative *F*^2^. The threshold expression of *F*^2^ > 2σ(*F*^2^) is used only for calculating *R*-factors(gt) *etc*. and is not relevant to the choice of reflections for refinement. *R*-factors based on *F*^2^ are statistically about twice as large as those based on *F*, and *R*- factors based on ALL data will be even larger.

### Fractional atomic coordinates and isotropic or equivalent isotropic displacement parameters (Å^2^)

**Table:**

	*x*	*y*	*z*	*U*_iso_*/*U*_eq_	
O2	0.2678 (3)	0.0244 (2)	0.16941 (11)	0.0232 (7)	
H2A	0.1904	0.0043	0.1841	0.035*	
O1	−0.2643 (3)	0.3748 (2)	0.32906 (12)	0.0237 (7)	
H1A	−0.1885	0.3958	0.3138	0.036*	
N2	−0.0279 (4)	0.4483 (3)	0.26652 (16)	0.0233 (9)	
N1	0.0312 (4)	−0.0498 (3)	0.23253 (16)	0.0212 (8)	
C31	0.2335 (5)	0.1227 (4)	0.14084 (16)	0.0204 (9)	
H31A	0.1542	0.1032	0.1140	0.025*	
C36	0.1502 (4)	0.2991 (3)	0.25476 (16)	0.0173 (9)	
H36A	0.1831	0.3043	0.2893	0.021*	
C11	−0.2249 (4)	0.2800 (4)	0.35820 (16)	0.0188 (9)	
H11A	−0.1447	0.3016	0.3843	0.023*	
C37	0.2122 (4)	0.2203 (3)	0.22438 (16)	0.0166 (9)	
H37A	0.2875	0.1718	0.2385	0.020*	
C15	−0.0345 (4)	0.0279 (3)	0.26470 (16)	0.0176 (9)	
C17	−0.2079 (5)	0.1796 (4)	0.27497 (16)	0.0208 (9)	
H17A	−0.2848	0.2270	0.2610	0.025*	
C35	0.0382 (5)	0.3716 (3)	0.23442 (16)	0.0196 (9)	
C16	−0.1464 (4)	0.0999 (4)	0.24464 (16)	0.0195 (9)	
H16A	−0.1803	0.0941	0.2102	0.023*	
C34	−0.0086 (5)	0.3614 (4)	0.18438 (17)	0.0221 (10)	
H34A	−0.0841	0.4097	0.1702	0.026*	
C14	0.0149 (5)	0.0395 (4)	0.31485 (17)	0.0216 (10)	
H14A	0.0908	−0.0085	0.3290	0.026*	
C13	−0.0459 (4)	0.1208 (4)	0.34459 (17)	0.0217 (10)	
H13A	−0.0092	0.1282	0.3787	0.026*	
C32	0.1667 (4)	0.2106 (3)	0.17363 (16)	0.0171 (9)	
C33	0.0537 (5)	0.2811 (4)	0.15445 (17)	0.0201 (9)	
H33A	0.0184	0.2744	0.1202	0.024*	
C21	0.3783 (5)	0.1564 (3)	0.11427 (16)	0.0189 (9)	
C12	−0.1594 (5)	0.1918 (4)	0.32555 (16)	0.0193 (9)	
C30	0.4344 (5)	0.0582 (4)	0.08343 (18)	0.0247 (10)	
H30A	0.4603	−0.0041	0.1063	0.030*	
H30B	0.3522	0.0339	0.0591	0.030*	
C8	−0.4950 (5)	0.2002 (4)	0.34928 (16)	0.0211 (10)	
H8A	−0.4571	0.1361	0.3307	0.025*	
H8B	−0.5245	0.2581	0.3245	0.025*	
C6	−0.6949 (5)	0.2663 (4)	0.40555 (18)	0.0285 (11)	
H6A	−0.7243	0.3243	0.3808	0.034*	
H6B	−0.7860	0.2458	0.4237	0.034*	
C28	0.5085 (5)	0.1941 (4)	0.15176 (16)	0.0196 (9)	
H28A	0.4754	0.2592	0.1707	0.023*	
H28B	0.5331	0.1345	0.1762	0.023*	
C27	0.6498 (5)	0.2230 (4)	0.12352 (16)	0.0213 (9)	
H27A	0.7332	0.2453	0.1484	0.026*	
C1	−0.3687 (4)	0.2436 (4)	0.38585 (16)	0.0200 (9)	
C26	0.7028 (5)	0.1259 (4)	0.09309 (17)	0.0248 (10)	
H26A	0.7301	0.0638	0.1159	0.030*	
H26B	0.7943	0.1468	0.0752	0.030*	
C7	−0.6353 (5)	0.1658 (4)	0.37817 (18)	0.0289 (11)	
H7A	−0.7164	0.1371	0.3539	0.035*	
C5	−0.5715 (5)	0.3100 (4)	0.44299 (18)	0.0292 (11)	
H5A	−0.6109	0.3755	0.4610	0.035*	
C10	−0.4314 (5)	0.3433 (4)	0.41430 (18)	0.0286 (11)	
H10A	−0.3515	0.3721	0.4384	0.034*	
H10B	−0.4596	0.4025	0.3900	0.034*	
C22	0.3428 (5)	0.2528 (4)	0.07819 (18)	0.0282 (11)	
H22A	0.3084	0.3167	0.0976	0.034*	
H22B	0.2592	0.2316	0.0538	0.034*	
C25	0.5754 (5)	0.0907 (4)	0.05480 (18)	0.0307 (11)	
H25A	0.6099	0.0264	0.0350	0.037*	
C29	0.6129 (5)	0.3197 (4)	0.08773 (19)	0.0307 (11)	
H29A	0.7044	0.3401	0.0698	0.037*	
H29B	0.5811	0.3843	0.1072	0.037*	
C23	0.4837 (5)	0.2858 (4)	0.04936 (18)	0.0313 (12)	
H23A	0.4578	0.3485	0.0262	0.038*	
C24	0.5362 (6)	0.1869 (5)	0.01922 (18)	0.0340 (12)	
H24A	0.6266	0.2073	0.0008	0.041*	
H24B	0.4545	0.1646	−0.0057	0.041*	
C4	−0.5269 (6)	0.2204 (5)	0.48089 (19)	0.0420 (14)	
H4A	−0.6164	0.1985	0.4995	0.050*	
H4B	−0.4479	0.2485	0.5055	0.050*	
C2	−0.3263 (5)	0.1538 (4)	0.42432 (19)	0.0335 (12)	
H2D	−0.2448	0.1809	0.4482	0.040*	
H2E	−0.2874	0.0886	0.4068	0.040*	
C9	−0.5907 (6)	0.0769 (4)	0.4164 (2)	0.0424 (14)	
H9A	−0.5532	0.0112	0.3989	0.051*	
H9B	−0.6804	0.0550	0.4350	0.051*	
C3	−0.4660 (6)	0.1212 (5)	0.4533 (2)	0.0456 (15)	
H3A	−0.4363	0.0626	0.4784	0.055*	
H1C	0.078 (5)	−0.107 (4)	0.2492 (17)	0.017 (11)*	
H2C	−0.081 (7)	0.496 (5)	0.248 (2)	0.043 (17)*	
H1B	−0.029 (6)	−0.075 (5)	0.211 (2)	0.033 (15)*	
H2B	0.039 (7)	0.482 (5)	0.289 (2)	0.057 (19)*	

### Atomic displacement parameters (Å^2^)

**Table:**

	*U*^11^	*U*^22^	*U*^33^	*U*^12^	*U*^13^	*U*^23^
O2	0.0184 (15)	0.0165 (16)	0.0354 (18)	0.0006 (12)	0.0083 (13)	0.0024 (13)
O1	0.0180 (15)	0.0158 (16)	0.0380 (18)	−0.0019 (12)	0.0085 (13)	0.0016 (13)
N2	0.0170 (19)	0.021 (2)	0.032 (2)	0.0003 (17)	0.0049 (17)	−0.0027 (18)
N1	0.0172 (19)	0.0143 (19)	0.032 (2)	0.0021 (16)	0.0040 (17)	−0.0019 (17)
C31	0.016 (2)	0.017 (2)	0.028 (2)	−0.0029 (17)	−0.0001 (17)	−0.0032 (18)
C36	0.0102 (19)	0.018 (2)	0.024 (2)	−0.0028 (16)	0.0021 (16)	0.0008 (17)
C11	0.0104 (19)	0.021 (2)	0.026 (2)	0.0003 (17)	0.0028 (16)	0.0023 (18)
C37	0.0107 (18)	0.012 (2)	0.027 (2)	−0.0021 (16)	0.0017 (16)	0.0017 (17)
C15	0.0091 (18)	0.017 (2)	0.027 (2)	−0.0040 (16)	0.0053 (16)	−0.0003 (17)
C17	0.0119 (19)	0.024 (2)	0.027 (2)	−0.0005 (17)	0.0023 (17)	0.0032 (19)
C35	0.016 (2)	0.014 (2)	0.029 (2)	−0.0037 (17)	0.0067 (17)	−0.0005 (18)
C16	0.015 (2)	0.019 (2)	0.025 (2)	−0.0030 (17)	0.0017 (17)	0.0002 (18)
C34	0.013 (2)	0.019 (2)	0.034 (3)	−0.0019 (17)	0.0007 (18)	0.0048 (19)
C14	0.0119 (19)	0.018 (2)	0.034 (3)	0.0002 (17)	0.0010 (17)	0.0039 (19)
C13	0.013 (2)	0.026 (2)	0.026 (2)	−0.0018 (18)	−0.0001 (17)	0.0014 (19)
C32	0.0097 (19)	0.015 (2)	0.027 (2)	−0.0025 (16)	0.0037 (16)	0.0008 (17)
C33	0.014 (2)	0.021 (2)	0.026 (2)	−0.0025 (17)	−0.0001 (17)	0.0016 (18)
C21	0.016 (2)	0.019 (2)	0.022 (2)	0.0009 (17)	0.0018 (17)	−0.0020 (18)
C12	0.014 (2)	0.021 (2)	0.023 (2)	−0.0050 (17)	0.0044 (17)	0.0010 (18)
C30	0.023 (2)	0.020 (2)	0.031 (3)	−0.0003 (19)	0.0063 (19)	−0.0067 (19)
C8	0.017 (2)	0.022 (2)	0.025 (2)	−0.0054 (18)	0.0045 (18)	−0.0031 (18)
C6	0.013 (2)	0.042 (3)	0.030 (3)	−0.006 (2)	0.0062 (18)	0.000 (2)
C28	0.016 (2)	0.022 (2)	0.021 (2)	−0.0045 (17)	0.0029 (17)	0.0001 (18)
C27	0.019 (2)	0.022 (2)	0.024 (2)	−0.0066 (18)	0.0048 (17)	−0.0020 (18)
C1	0.0121 (19)	0.025 (2)	0.023 (2)	−0.0019 (18)	0.0014 (16)	0.0013 (19)
C26	0.020 (2)	0.023 (2)	0.032 (3)	0.0020 (18)	0.0071 (18)	−0.001 (2)
C7	0.022 (2)	0.032 (3)	0.033 (3)	−0.010 (2)	0.006 (2)	−0.005 (2)
C5	0.021 (2)	0.036 (3)	0.032 (3)	−0.005 (2)	0.0091 (19)	−0.013 (2)
C10	0.018 (2)	0.038 (3)	0.030 (3)	−0.006 (2)	0.0040 (19)	−0.010 (2)
C22	0.022 (2)	0.034 (3)	0.029 (2)	0.006 (2)	0.0035 (19)	0.009 (2)
C25	0.030 (3)	0.032 (3)	0.031 (3)	−0.003 (2)	0.008 (2)	−0.006 (2)
C29	0.031 (3)	0.020 (2)	0.043 (3)	−0.007 (2)	0.018 (2)	0.001 (2)
C23	0.031 (3)	0.037 (3)	0.027 (3)	0.003 (2)	0.010 (2)	0.012 (2)
C24	0.030 (3)	0.051 (3)	0.021 (2)	−0.002 (2)	0.007 (2)	0.001 (2)
C4	0.024 (3)	0.077 (4)	0.025 (3)	0.000 (3)	0.006 (2)	0.001 (3)
C2	0.028 (3)	0.041 (3)	0.032 (3)	0.012 (2)	0.007 (2)	0.014 (2)
C9	0.041 (3)	0.027 (3)	0.062 (4)	0.000 (2)	0.030 (3)	0.009 (3)
C3	0.036 (3)	0.056 (4)	0.046 (3)	0.008 (3)	0.012 (2)	0.023 (3)

### Geometric parameters (Å, °)

**Table:**

O2—C31	1.440 (5)	C8—H8B	0.9900
O2—H2A	0.8400	C6—C5	1.530 (6)
O1—C11	1.422 (5)	C6—C7	1.530 (7)
O1—H1A	0.8400	C6—H6A	0.9900
N2—C35	1.411 (6)	C6—H6B	0.9900
N2—H2C	0.88 (6)	C28—C27	1.529 (6)
N2—H2B	0.91 (7)	C28—H28A	0.9900
N1—C15	1.419 (5)	C28—H28B	0.9900
N1—H1C	0.91 (5)	C27—C26	1.519 (6)
N1—H1B	0.83 (6)	C27—C29	1.537 (6)
C31—C32	1.518 (6)	C27—H27A	1.0000
C31—C21	1.547 (6)	C1—C2	1.529 (6)
C31—H31A	1.0000	C1—C10	1.547 (6)
C36—C37	1.384 (6)	C26—C25	1.537 (6)
C36—C35	1.410 (6)	C26—H26A	0.9900
C36—H36A	0.9500	C26—H26B	0.9900
C11—C12	1.514 (6)	C7—C9	1.522 (7)
C11—C1	1.562 (6)	C7—H7A	1.0000
C11—H11A	1.0000	C5—C4	1.523 (8)
C37—C32	1.393 (6)	C5—C10	1.540 (6)
C37—H37A	0.9500	C5—H5A	1.0000
C15—C14	1.389 (6)	C10—H10A	0.9900
C15—C16	1.402 (6)	C10—H10B	0.9900
C17—C16	1.390 (6)	C22—C23	1.546 (6)
C17—C12	1.398 (6)	C22—H22A	0.9900
C17—H17A	0.9500	C22—H22B	0.9900
C35—C34	1.378 (6)	C25—C24	1.533 (7)
C16—H16A	0.9500	C25—H25A	1.0000
C34—C33	1.391 (6)	C29—C23	1.544 (7)
C34—H34A	0.9500	C29—H29A	0.9900
C14—C13	1.390 (6)	C29—H29B	0.9900
C14—H14A	0.9500	C23—C24	1.530 (7)
C13—C12	1.396 (6)	C23—H23A	1.0000
C13—H13A	0.9500	C24—H24A	0.9900
C32—C33	1.391 (6)	C24—H24B	0.9900
C33—H33A	0.9500	C4—C3	1.524 (8)
C21—C22	1.536 (6)	C4—H4A	0.9900
C21—C30	1.545 (6)	C4—H4B	0.9900
C21—C28	1.549 (6)	C2—C3	1.539 (7)
C30—C25	1.543 (6)	C2—H2D	0.9900
C30—H30A	0.9900	C2—H2E	0.9900
C30—H30B	0.9900	C9—C3	1.533 (8)
C8—C1	1.532 (6)	C9—H9A	0.9900
C8—C7	1.548 (6)	C9—H9B	0.9900
C8—H8A	0.9900	C3—H3A	1.0000
			
C31—O2—H2A	109.5	C28—C27—C29	109.2 (4)
C11—O1—H1A	109.5	C26—C27—H27A	109.1
C35—N2—H2C	109 (4)	C28—C27—H27A	109.1
C35—N2—H2B	115 (4)	C29—C27—H27A	109.1
H2C—N2—H2B	112 (6)	C2—C1—C8	108.8 (4)
C15—N1—H1C	114 (3)	C2—C1—C10	108.2 (4)
C15—N1—H1B	114 (4)	C8—C1—C10	108.3 (3)
H1C—N1—H1B	109 (5)	C2—C1—C11	110.0 (3)
O2—C31—C32	111.0 (3)	C8—C1—C11	112.2 (3)
O2—C31—C21	107.8 (3)	C10—C1—C11	109.1 (4)
C32—C31—C21	115.5 (3)	C27—C26—C25	109.6 (4)
O2—C31—H31A	107.4	C27—C26—H26A	109.8
C32—C31—H31A	107.4	C25—C26—H26A	109.8
C21—C31—H31A	107.4	C27—C26—H26B	109.8
C37—C36—C35	119.9 (4)	C25—C26—H26B	109.8
C37—C36—H36A	120.0	H26A—C26—H26B	108.2
C35—C36—H36A	120.0	C9—C7—C6	109.4 (4)
O1—C11—C12	110.5 (3)	C9—C7—C8	109.8 (4)
O1—C11—C1	107.8 (3)	C6—C7—C8	108.9 (4)
C12—C11—C1	114.4 (4)	C9—C7—H7A	109.6
O1—C11—H11A	108.0	C6—C7—H7A	109.6
C12—C11—H11A	108.0	C8—C7—H7A	109.6
C1—C11—H11A	108.0	C4—C5—C6	109.4 (4)
C36—C37—C32	121.5 (4)	C4—C5—C10	109.3 (4)
C36—C37—H37A	119.3	C6—C5—C10	109.2 (4)
C32—C37—H37A	119.3	C4—C5—H5A	109.6
C14—C15—C16	118.7 (4)	C6—C5—H5A	109.6
C14—C15—N1	122.0 (4)	C10—C5—H5A	109.6
C16—C15—N1	119.2 (4)	C5—C10—C1	110.7 (4)
C16—C17—C12	121.6 (4)	C5—C10—H10A	109.5
C16—C17—H17A	119.2	C1—C10—H10A	109.5
C12—C17—H17A	119.2	C5—C10—H10B	109.5
C34—C35—C36	118.8 (4)	C1—C10—H10B	109.5
C34—C35—N2	122.1 (4)	H10A—C10—H10B	108.1
C36—C35—N2	119.0 (4)	C21—C22—C23	111.4 (4)
C17—C16—C15	120.1 (4)	C21—C22—H22A	109.3
C17—C16—H16A	119.9	C23—C22—H22A	109.3
C15—C16—H16A	119.9	C21—C22—H22B	109.3
C35—C34—C33	120.6 (4)	C23—C22—H22B	109.3
C35—C34—H34A	119.7	H22A—C22—H22B	108.0
C33—C34—H34A	119.7	C24—C25—C26	109.2 (4)
C15—C14—C13	120.6 (4)	C24—C25—C30	109.9 (4)
C15—C14—H14A	119.7	C26—C25—C30	108.9 (4)
C13—C14—H14A	119.7	C24—C25—H25A	109.6
C14—C13—C12	121.5 (4)	C26—C25—H25A	109.6
C14—C13—H13A	119.2	C30—C25—H25A	109.6
C12—C13—H13A	119.2	C27—C29—C23	109.2 (4)
C33—C32—C37	117.8 (4)	C27—C29—H29A	109.8
C33—C32—C31	121.0 (4)	C23—C29—H29A	109.8
C37—C32—C31	121.1 (4)	C27—C29—H29B	109.8
C32—C33—C34	121.3 (4)	C23—C29—H29B	109.8
C32—C33—H33A	119.4	H29A—C29—H29B	108.3
C34—C33—H33A	119.4	C24—C23—C29	108.9 (4)
C22—C21—C30	108.6 (4)	C24—C23—C22	109.2 (4)
C22—C21—C31	110.0 (3)	C29—C23—C22	108.9 (4)
C30—C21—C31	109.4 (3)	C24—C23—H23A	109.9
C22—C21—C28	107.2 (4)	C29—C23—H23A	109.9
C30—C21—C28	108.9 (3)	C22—C23—H23A	109.9
C31—C21—C28	112.6 (3)	C23—C24—C25	109.9 (4)
C13—C12—C17	117.4 (4)	C23—C24—H24A	109.7
C13—C12—C11	121.2 (4)	C25—C24—H24A	109.7
C17—C12—C11	121.4 (4)	C23—C24—H24B	109.7
C25—C30—C21	110.6 (4)	C25—C24—H24B	109.7
C25—C30—H30A	109.5	H24A—C24—H24B	108.2
C21—C30—H30A	109.5	C5—C4—C3	109.5 (4)
C25—C30—H30B	109.5	C5—C4—H4A	109.8
C21—C30—H30B	109.5	C3—C4—H4A	109.8
H30A—C30—H30B	108.1	C5—C4—H4B	109.8
C1—C8—C7	110.5 (4)	C3—C4—H4B	109.8
C1—C8—H8A	109.6	H4A—C4—H4B	108.2
C7—C8—H8A	109.6	C1—C2—C3	110.2 (4)
C1—C8—H8B	109.6	C1—C2—H2D	109.6
C7—C8—H8B	109.6	C3—C2—H2D	109.6
H8A—C8—H8B	108.1	C1—C2—H2E	109.6
C5—C6—C7	109.8 (4)	C3—C2—H2E	109.6
C5—C6—H6A	109.7	H2D—C2—H2E	108.1
C7—C6—H6A	109.7	C7—C9—C3	109.3 (4)
C5—C6—H6B	109.7	C7—C9—H9A	109.8
C7—C6—H6B	109.7	C3—C9—H9A	109.8
H6A—C6—H6B	108.2	C7—C9—H9B	109.8
C27—C28—C21	110.2 (3)	C3—C9—H9B	109.8
C27—C28—H28A	109.6	H9A—C9—H9B	108.3
C21—C28—H28A	109.6	C4—C3—C9	109.1 (4)
C27—C28—H28B	109.6	C4—C3—C2	110.4 (5)
C21—C28—H28B	109.6	C9—C3—C2	109.5 (4)
H28A—C28—H28B	108.1	C4—C3—H3A	109.2
C26—C27—C28	111.4 (4)	C9—C3—H3A	109.2
C26—C27—C29	109.0 (4)	C2—C3—H3A	109.2

### Hydrogen-bond geometry (Å, °)

**Table:**

*D*—H···*A*	*D*—H	H···*A*	*D*···*A*	*D*—H···*A*
O2—H2A···N1	0.84	2.06	2.890 (5)	168
O1—H1A···N2	0.84	2.04	2.876 (5)	171
N1—H1B···O1^i^	0.83 (6)	2.15 (6)	2.941 (5)	162 (5)
N2—H2B···O2^ii^	0.91 (7)	2.05 (7)	2.932 (5)	163 (6)

Symmetry codes: (i) −*x*−1/2, *y*−1/2, −*z*+1/2; (ii) −*x*+1/2, *y*+1/2, −*z*+1/2.
